# Effect of Dietary Vitamin A on Reproductive Performance and Immune Response of Broiler Breeders

**DOI:** 10.1371/journal.pone.0105677

**Published:** 2014-08-22

**Authors:** Jianmin Yuan, Abdelfatah Rashad Roshdy, Yuming Guo, Yongwei Wang, Shuangshuang Guo

**Affiliations:** 1 State Key Lab of Animal Nutrition, College of Animal Science and Technology, China Agricultural University, Beijing, China; 2 Faculty of Environmental Agricultural Sciences, Suez Canal University, Ismaïlia, Egypt; University of Pittsburgh, United States of America

## Abstract

The effects of dietary vitamin A supplementation on reproductive performance, liver function, fat-soluble vitamin retention, and immune response were studied in laying broiler breeders. In the first phase of the experiment, 1,120 Ross-308 broiler breeder hens were fed a diet of corn and soybean meal supplemented with 5,000 to 35,000 IU/kg vitamin A (retinyl acetate) for 20 weeks. In the second phase, 384 Ross-308 broiler breeder hens were fed the same diet supplemented with 5,000 to 135,000 IU/kg vitamin A (retinyl acetate) for 24 weeks. The hens' reproductive performance, the concentrations of vitamins A and E in liver and egg yolk, liver function, mRNA expression of vitamin D receptor in duodenal mucosa, antibody titers against Newcastle disease virus vaccine, and T-cell proliferation responses were evaluated. Supplementation of vitamin A at levels up to and including 35,000 IU/kg did not affect reproductive performance and quadratically affected antibody titer to Newcastle disease virus vaccine (*p*<0.05). Dietary addition of vitamin A linearly increased vitamin A concentration in liver and yolk and linearly decreased α-, γ-, and total tocopherol concentration in yolk (*p*<0.01) and α-tocopherol in liver (*p*<0.05). Supplementation of vitamin A at doses of 45,000 IU/kg and above significantly decreased egg weight, yolk color, eggshell thickness and strength, and reproductive performance. Dietary vitamin A significantly increased mRNA expression of vitamin D receptor in duodenal mucosa (*p*<0.05), increased aspartate amino transferase activity, and decreased total bilirubin concentration in serum. Supplementation of vitamin A at 135,000 IU/kg decreased the proliferation of peripheral blood lymphocytes (*p*<0.05). Therefore, the maximum tolerable dose of vitamin A for broiler breeders appears to be 35,000 IU/kg, as excessive supplementation has been shown to impair liver function, reproductive performance, and immune response.

## Introduction

Vitamin A is an essential micronutrient throughout the life cycle. Dietary vitamin A deficiency decreased performance, caused infertility or impaired reproduction [1], and depressed T-lymphocyte responses and antibody production in poultry [2], [3]. Vitamin A supplementation of the diet could prevent inhibition of growth performance in poultry that would otherwise experience a deficiency of this vitamin. The National Research Council (NRC) (1987) [4] recommended a dosage of 3,000 IU/kg of vitamin A in poultry and indicated that the maximum tolerable dose for laying hens is 40,000 IU/kg. Previous studies showed that diets supplemented with high levels of vitamin A above the NRC recommendation had no significant effect on the laying performance of layer hens under normal conditions [5], [6]. However, diets that included higher doses of vitamin A than the NRC recommendation improved the laying performance of heat-stressed hens [7].

Today, broiler breeding companies recommend 10,000 IU/kg vitamin as the requirement level of broilers or broiler breeders [8]. As a fat-soluble vitamin, vitamin A can be efficiently stored in the liver and yolk of birds, and it plays a role in both the biology of the adult bird and embryo development [9]. However, excess amounts of vitamin A retained in the body may be toxic to poultry.Vitamin A is easily destroyed in feed processing and storage. Usually, 10,000–15,000 IU/kg vitamin A is supplemented in broiler or broiler breeder commercial feed. Higher levels of vitamin A (210,000 to 410,000 IU/kg) decreased egg production, size, and hatchability in laying hens [10]. Vitamin A may interfere with the absorption of other fat-soluble vitamins and is known to decrease α-tocopherol deposition in egg yolk [11], [12]. Excessive vitamin A intake resulted in congenital malformations during embryonic development [1] and also had a toxic effect on the liver in broilers [13]. Excessive supplementation with vitamin A might increase the risk of bone fractures by depleting vitamin D [14], and it has a detrimental effect on the immune function of birds [15]. However, Veltmann et al. reported that birds could tolerate as much as 30 times the recommended level of vitamin A without showing compromised performance or damage to skeletal development, as measured by bone ash [16].

Although many studies have examined the effects of vitamin A on immunity, growth, and development in broilers and layers, few studies have explored the effects of high levels of dietary vitamin A supplementation on broiler breeder hens. Veltmann and Jensen compared vitamin A toxicity in Single Comb White Leghorn chicks, broilers, and turkey poults and found that a differential response to vitamin A toxicosis existed within breeds and across species [17]. The effect of high levels of dietary vitamin A supplementation on broiler breeder hens is unknown. In the current study, experiment 1 was designed to evaluate the effects of high levels of dietary supplementation of vitamin A on reproductive performance, vitamin A and E concentrations in the yolk and in the liver, and antibody production of broiler breeder hens. Experiment 2 was used to study much higher levels of dietary supplementation of vitamin A on reproductive performance and to explain the mechanism of vitamin A's effects on vitamin D absorption, liver function, and the T-cell proliferation response.

## Material and Methods

All animal studies were approved by the Animal Care and Use Committee at China Agricultural University before initiation.The birds were sacrificed by intracardiac injection of sodium pentobarbital.

### Experiment 1

A total of 1,120 36-week-old Ross-308 broiler breeder hens were randomly allotted to seven treatments with eight replicates, with 20 hens assigned to each replicate. The hens' diets were supplemented with 5,000; 10,000; 15,000; 20,000; 25,000; 30,000; or 35,000 IU/kg vitamin A (retinyl acetate) in the diet. The experiment lasted for 20 weeks. The basal diet was formulated according to the recommendation of the NRC (1994) [18] (Table 1). The numbers of normal eggs, broken eggs, abnormal eggs, and dead hens were recorded every day.

**Table 1:** Composition of diets and nutrient levels of broiler breeder experiment.

Ingredients	Composition (%)	Nutrient	Calculated analysis
Corn	63.45	ME (MCal/Kg)	2.81
Soybean meal	24.40	CP (%)	17.31
Limestone	8.20	Lys (%)	0.85
Soybean oil	2.00	Met (%)	0.38
Dicalcium Phosphate	0.96	Met+Cys (%)	0.67
DL-Methionine	0.10	Thr (%)	0.66
Choline chloride(50%)	0.12	Trp(%)	0.21
NaCl	0.35	Ca (%)	3.20
Flavomycin(4%)	0.02	AP (%)	0.30
Ethoxyquin(33%)	0.03		
Phytase(2500 U/g)	0.04		
Mineral premix	0.20		
Vitamin premix	0.13		

Mineral premix provided per kilogram of diet: Mn, 100 mg; Fe, 80 mg; Zn, 75 mg; Cu, 8 mg; I, 0.35 mg; Se, 0.30 mg.

Vitamin premix provided per kilogram of diet: vitamin D_3_, 2,500 IU; vitamin E, 30 IU; vitamin K_3_, 2.65 mg; vitamin B_1_, 2 mg; vitamin B_2_, 6 mg; vitamin B_12_, 0.025 mg; biotin, 0.0325 mg; folic acid, 1.25 mg; pantothenic acid, 12 mg; niacin, 50 mg.

At 4, 8, 12, 16, and 20 weeks, laid eggs were collected and hatched, and egg quality measurements were performed on three fresh eggs from each replicate. At 12 and 20 weeks, the amounts of vitamins A and E in egg yolks were measured. Every four weeks, a blood sample was collected from the brachial vein of one bird per replicate and centrifuged (3000×g for 10 min) to obtain serum for an antibody titer to Newcastle disease virus (NDV) measurement. At 12 and 20 weeks, one bird per replicate was killed, and the amounts of vitamins A and E in the liver were measured by high-performance liquid chromatography.

### Experiment 2

A total of 384 31-week-old Ross-308 broiler breeder hens were divided into four treatments with six replicates, with 16 hens assigned to each replicate. The hens' diets were supplemented with 5,000; 15,000; 45,000; and 135,000 IU/kg vitamin A (retinyl acetate). The experiment lasted for 24 weeks. The basal diet was the same as in Experiment 1.

Performance was measured as in Experiment 1. At the 12-week and 24-week marks of the experiment, laid eggs were collected and hatched. Every 12 weeks (at 42 and 55 weeks of age), egg quality measurements were performed on three fresh eggs from each replicate. Every 12 weeks of the experiment, a blood sample was collected from the brachial vein of one bird per replicate to measure peripheral blood lymphocyte proliferation, total bilirubin (TBIL), and aspartate amino transferase (AST).

At the end of the experiment, one bird per replicate was killed, and the intestinal mucosa of the duodenum was scraped, washed by cold phosphate buffer saline (PBS), frozen in liquid nitrogen, and stored at −80°C to measure mRNA expression of vitamin D receptor (VDR).

### Housing and management

Experimental hens were raised in a temperature-controlled broiler breeder house. All the birds had free access to feed and water during the entire period. At the start of the experiment, 160 g of feed was provided per bird, per day. From 41 weeks of age, feed was reduced by 0.5 g every week to prevent the hens from developing obesity. The hens underwent artificial insemination every five days. The male breeders used for artificial insemination were kept in separate quarters from the laying hens, and were fed the commercial breeder diet supplemented with 12,000 IU/kg vitamin A. The light regime was 16-h light:8-h dark. The broiler breeders were vaccinated against inactivated NDV vaccine at 20 weeks of age by receiving an injection in the back of the neck, and they received live NDV vaccine at 32 weeks of age by spray.

### Egg quality

Eggshell strength was determined using Eggshell Force Gauge (Robotmation Co. Ltd., Tokyo, Japan), and albumen height, Hugh unit, and yolk color were evaluated using the Egg Multi Tester EMT-5200 (Robotmation Co., Ltd.). Eggshell thickness without inner and outer membranes was measured at the blunt, equatorial, and sharp regions.

### Egg hatchability

Eggs were stored at 7°C before incubated. The temperature was 37.8°C and 50% relative humidity. On the 17th day, the eggs were candled to determine the number of infertile eggs and the number of early dead embryos. After 21 days of incubation, the number of healthy chicks was recorded.

### Antibody response to NDV

An antibody titer was determined by the hemagglutination inhibition technique [19]. The titer was expressed as log2 of the highest dilution based on total agglutination.

### Concentrations of vitamins A and E in liver and yolk

Liver samples were homogenized and saponified, and extraction of vitamins A and E from the yolk and liver was performed as described by Boily et al. [20]. The concentration of vitamins A and E was analyzed by the high-performance liquid chromatography method of Catignani et al. [21].

### Peripheral T-lymphocyte proliferation

A 3-[4,5-dimethylthiazol]-2,5-diphenyltetrazolium bromide (MTT, Sigma Chemical Co., St. Louis, MO) assay was used to determine the peripheral blood lymphocyte proliferation response at 42 and 55 weeks of age in Experiment 2. Six healthy chickens (one per replicate) were randomly chosen from each treatment. Heparinized blood samples were collected from the wing vein and each blood sample was added to isometric lymphocyte separation medium (density  = 1.077; HaoYang Biological Manufacture Co., Ltd., Tianjin, China). Lymphocytes were isolated after 30 min, and centrifugation proceeded at 1,006 µ*g* at 4°C. The lymphocyte fraction was collected from the interface and washed three times with RPMI 1640 (Invitrogen Corp., Grand Island, NY) incomplete culture medium. Lymphocytes were then resuspended in 2 mL of RPMI 1640 complete culture medium supplemented with 5% (vol/vol) of fetal calf serum, 0.5% penicillin (final concentration, 100 U/mL), 0.5% streptomycin (final concentration, 100 µg/mL), and 1% N-(2-hydroxyethyl)-piperazine-N-2-ethane-sulfonic acid (HEPES, final concentration, 24 m*M*; Amresco 0511, Amresco Inc., Cleveland, OH).

Cells were detected by trypan blue dye exclusion and counted to adjust the density of the cells to 1×10^7^cells per milliliter of culture medium. One hundred microliters of cell suspension and the lymphocyte mitogen concanavalin A (Con A, Sigma Chemical Co.) or LPS (Sigma Chemical Co.) was added to a 96-well microtiter plate (Costar 3599, Corning, Inc., Corning, NY) to provide a final concentration of 45 µg/mL (Con A) or 25 µg/mL (LPS). Cells then were stored at 37°C with 5% CO_2_ in an incubator (MCO-18AIC CO_2_ incubator, Sanyo Electric Biomedical Co. Ltd., Tokyo, Japan). After 68 h, 15 µL of 5 mg/mL MTT was added to each well and the plates were incubated for another 4 h. Subsequently, 100 µL of 10% sodium dodecyl sulfate dissolved in 0.04 mol/L HCl solution was added to each well to lyse the cells and solubilize the MTT crystals. Finally, plates were read using an automated ELISA reader (model 550 Microplate Reader, Bio-Rad Pacific Ltd., Hong Kong, China) at 570 nm [22].

### Serum biochemistry

Total bilirubin (TBIL) and aspartate amino transferase (AST) were quantified spectrophotometrically, using commercial kits as manuscript guide (Jian Cheng Bioengineering Institute, Nanjing, China).

### Vitamin D receptor mRNA expression

Total RNA was extracted from the duodenal mucosa of the hens at the end of the experiment, according to SV Total RNA Isolation System instructions (Z3100; Promega, Madison, WI, USA), and resuspended in diethylpyrocarbonate-treated water. The concentration and quality of the total RNA were determined by absorbance at 260 nm and by agarose gel electrophoresis, respectively. Then 1.0 µg of total RNA was reversed into single-stranded cDNA with Avian Myeloblastosis Virus Reverse Transcriptase (Promega, Madison, WI, USA) using an oligo (dT) 15 primer in the presence of recombinant RNase in Ribonuclease Inhibitor (A3500; Promega, Madison, WI, USA).

Real-time polymerase chain reaction (PCR) analysis of vitamin D (1,25-dihydroxyvitamin D_3_) receptor mRNA was performed with β-actin as the internal control standard. The primers used and PCR product lengths are listed in Table 2. Real-time PCR was conducted on an ABI 7500 Fluorescent Quantitative PCR system (Applied Biosystems, Bedford, MA, USA) using the RealSuper Mixture with Rox (CW0767; CWbio Company, Sandringham, UK). The protocol used was as follows: 95°C for 4 min; 40 cycles of 95°C for 15 sec, 60°C for 60 sec; and 60°C for 95 sec for melting curve analysis. Each gene was amplified in triplicate. Standard curves were run to determine the amplification efficiency. The results were expressed as the ratio of the target gene mRNA to β-actin mRNA. The 2^–ΔΔCt^ method was used to calculate the expression of the target gene, as previously described [23].

**Table 2:** Primers used for the determination of vitamin D receptor protein (VDR) and calcium binding protein (Calbindin).

Primer name	Genebank accession	Orientation	Primer sequences (5′ to 3′)	Amplification products (bp)
VDR	NM_205098. 1	Forward	TGGGAAAGGCGATGCTGATG	168
		Reverse	TGATTGTGGTGGCAGTAGTG	
β-actin	NM_205518. 1	Forward	AACACCCACACCCCTGTGAT	100
		Reverse	TGAGTCAAGCGCCAAAAGAA	

### Statistical analysis

The results were analyzed by one-way ANOVA and GLM of SPSS 15.0 for Windows [24]. Linear and quadratic effects were tested and considered significant at *p*<0.05.

## Results

### Laying performance of broiler breeders

Different dietary levels of vitamin A supplementation had no significant effect on the percentage of normal eggs, abnormal eggs, broken eggs or on the mortality of broiler breeders in either experiment(data not shown). Average egg weight was not affected by different levels of vitamin A in Experiment 1. However, average egg weight from week 9 to week 16 linearly decreased with increasing vitamin A levels in Experiment 2 (Table 3).

**Table 3:** Average weight of normal eggs(g).

Vitamin A Treatment (IU/kg)	Weeks 1–8	Weeks 9–16	Weeks 17–24	Weeks 1–24
A (5,000)	62.65	69.65	74.96^b^	67.62
B (15,000)	62.60	69.17	73.63^ab^	67.23
C (45,000)	62.18	69.09	73.25^a^	67.05
D (135,000)	62.02	69.01	72.86^a^	66.82
SEM	0.243	0.202	0.272	0.173
*p*-value				
Combined	0.755	0.684	0.021	0.433
Linear	0.308	0.285	0.004	0.110
Quadratic	0.912	0.625	0.309	0.822

a–b Within a column, values not sharing a common superscript letter are significantly different (P<0.05).

### Egg quality

Different supplemental levels of vitamin A did not affect albumin height, Hugh unit, yolk color, eggshell strength, or eggshell thickness in Experiment 1 (data not shown). They also did not affect albumin height or Hugh unit in Experiment 2. However, the yolk color of eggs in week 12 and week 24 linearly decreased. The eggshell thickness (*p*<0.05) and strength (*p*<0.10) in week 24 was quadratically affected by increasing vitamin A levels in Experiment 2 (Table 4).

**Table 4:** Egg quality.

VA Treatment (IU/kg)	Week 12 of experiment			Week 24 of experiment		
	Yolk color	Eggshell thickness (µm)	Strength (kg/cm^2^)	Yolk color	Eggshell thickness (µm)	Eggshell strength (kg/cm^2^)
A (5,000)	8.72^c^	0.34	3.34	9.44^c^	0.36^b^	3.66
B (15,000)	8.33^c^	0.33	3.41	8.96^bc^	0.34^a^	3.19
C (45,000)	7.72^b^	0.34	3.31	8.37^b^	0.34^a^	3.47
D (135,000)	6.67^a^	0.33	3.26	6.18^a^	0.35^a^	3.64
SEM	0.132	0.001	0.091	0.202	0.002	0.104
P -value						
Combined	<0.001	0.843	0.949	<0.001	0.034	0.281
Linear	<0.001	0.906	0.906	0.007	0.036	0.807
Quadratic	0.081	0.660	0.781	0.239	0.034	0.099

a–c Within a column, values not sharing a common superscript letter are significantly different (P<0.05).

### Egg hatchability

Vitamin A levels did not affect the hatchability of hatched or fertile eggs in Experiment 1 (data not shown) or the hatchability of hatched or fertile eggs in week 12 in Experiment 2 (Table 5). However, the hatchability of hatched or fertile eggs at week 24 significantly decreased with increasing vitamin A levels (*p*<0.05), and higher levels of supplemental vitamin A tended to linearly decrease the number of healthy chicks at week 24 (*p*<0.10) in Experiment 2 (Table 5).

**Table 5:** Fertility and hatchability at week 12 and week 24 of the experiment.

VA Treatment (IU/kg)	Hatched egg %		Fertile egg %		Healthy chicks (% of Hatched Eggs)	
	Week 12	Week 24	Week 12	Week 24	Week 12	Week 24
A (5,000)	87.25	81.18^b^	93.10	88.01^b^	97.84	96.40
B (15,000)	86.62	81.19^b^	95.89	92.30^b^	98.17	96.69
C (45,000)	90.11	75.21^ab^	96.55	85.82^ab^	97.95	96.33
D (135,000)	87.16	68.51^a^	92.80	79.13^a^	98.15	93.00
SEM	1.113	1.683	0.812	1.563	0.323	0.654
*p*-value						
(Combined)	0.705	0.011	0.254	0.014	0.985	0.133
Linear	0.757	0.002	0.975	0.009	0.833	0.065
Quadratic	0.619	0.234	0.052	0.046	0.937	0.138

a–b Within a column, values not sharing a common superscript letter are significantly different (P<0.05).

### Concentration of vitamins A and E in liver and yolk

The vitamin A concentration in liver and yolk (*p*<0.001) linearly increased with increasing vitamin A levels in Experiment 1, both in week 12 and week 20 (Table 6). However, increasing levels of vitamin A linearly decreased the concentrations of α-, γ-, and total tocopherol in the yolk (*p*<0.01) and α-tocopherol in the liver (*p*<0.05). Total vitamin E content in the liver tended to decrease with increased vitamin A supplementation (*p*<0.10). The concentrations of δ-tocopherol in yolk and γ- and δ-tocopherol in the liver linearly increased with increasing vitamin A levels (Table 7).

**Table 6:** Vitamin A concentration in liver and yolk (mg/100 g).

	Vitamin A in liver (mg/100 g)		Vitamin A in yolk (mg/100 g)	
Treatment	Week 12	Week 20	Week 12	Week 20
A (5,000 IU/kg)	45.84^a^	41.24^a^	0.68^a^	0.58^a^
B (10,000 IU/kg)	54.80^ab^	54.70^ab^	0.69^a^	0.64^a^
C (15,000 IU/kg)	86.36^bc^	83.26^abc^	0.72^ab^	0.76^bc^
D (20,000 IU/kg)	82.48^bc^	94.98^bc^	0.83^bc^	0.73^b^
E (25,000 IU/kg)	110.80^c^	121.36^cd^	0.87^c^	0.85^cd^
F (30,000 IU/kg)	160.40^d^	144.56^d^	0.95^c^	0.83^cd^
G (35,000 IU/kg)	163.40^d^	195.20^e^	0.85^bc^	0.87^d^
SEM	8.272	9.893	0.023	0.022
*p*-value				
(Combined)	<0.001	<0.001	<0.001	<0.001
Linear	<0.001	<0.001	<0.001	<0.001
Quadratic	0.201	0.176	0.220	0.066

a–e Within a column, values not sharing a common superscript letter are significantly different (P<0.05).

**Table 7:** Vitamin E concentration in liver and yolk (mg/100 g).

	Yolk				Liver			
Treatment	α-VE (mg/100 g)	γ-VE (mg/100 g)	δ-VE (mg/100 g)	VE (mg/100 g)	α-VE mg/100 g)	γ-VE (mg/100 g)	δ-VE (mg/100 g)	VE (mg/100 g)
A (5,000 IU/kg)	14.62^c^	0.96^c^	0.11^a^	15.68^b^	2.42^d^	0.53^ab^	0.07^a^	3.02
B (10,000 IU/kg)	11.74^bc^	0.80^bc^	0.16^ab^	12.7^ab^	1.38^abcd^	0.45^a^	0.11^a^	1.93
C (15,000 IU/kg)	10.25^ab^	0.81^bcd^	0.21^b^	11.26^a^	1.69^bcd^	0.78^abc^	0.19^ab^	2.67
D (20,000 IU/kg)	10.36^ab^	0.82^cd^	0.29^c^	11.48^a^	2.01^cd^	1.01^abc^	0.25^ab^	3.28
E (25,000 IU/kg)	8.05^ab^	0.61^a^	0.34^cd^	9.02^a^	1.11^abc^	1.19^bc^	0.36^b^	2.62
F (30,000 IU/kg)	8.29^ab^	0.68^abc^	0.38^de^	9.36^a^	0.70^ab^	1.30^c^	0.24^ab^	2.23
G (35,000 IU/kg)	7.51^a^	0.62^ab^	0.45^e^	8.58^a^	0.37^a^	0.60^ab^	0.31^ab^	1.28
SEM	0.591	0.032	0.023	0.591	0.172	0.092	0.033	0.202
*p*-value								
(Combined)	0.006	0.003	<0.001	0.007	0.005	0.036	0.127	0.132
Linear	<0.001	<0.001	<0.001	<0.001	<0.001	0.042	0.008	0.091
Quadratic	0.226	0.508	0.845	0.230	0.459	0.030	0.283	0.116

a–e Within a column, values not sharing a common superscript letter are significantly different (P<0.05).

### Serum antibody against NDV

Different levels of supplemental vitamin A did not affect anti-NDV antibody titer at week 4 or week 8. However, vitamin A levels quadratically affected NDV antibody production at week 12 (Table 8) (*p*<0.05).

**Table 8:** Antibody response to Newcastle disease virus (NDV).

Treatment	Week 4	Week 8	Week 12	Week 16	Week 20
A (5,000 IU/kg)	9.80	8.25	6.33^a^	9.25^b^	6.80
B (10,000 IU/kg)	8.80	7.60	7.00^a^	8.00^ab^	7.20
C (15,000 IU/kg)	9.80	7.00	6.67^a^	8.25^ab^	7.40
D (20,000 IU/kg)	9.20	6.80	8.33^bc^	6.75^a^	7.20
E (25,000 IU/kg)	9.40	7.20	8.75^c^	6.60^a^	7.60
F (30,000 IU/kg)	8.80	7.40	7.33^ab^	6.80^a^	7.80
G (35,000 IU/kg)	9.60	6.80	6.75^a^	7.40^a^	8.00
SEM	0.136	0.190	0.223	0.233	0.138
*p*-value					
(Combined)	0.195	0.478	0.003	0.012	0.282
Linear	0.143	0.660	0.002	0.142	0.975
Quadratic	0.108	0.731	0.044	0.603	0.936

a–c Within a column, values not sharing a common superscript letter are significantly different (P<0.05).

### Peripheral blood lymphocyte proliferation

Dietary vitamin A levels had a significant effect on peripheral blood lymphocyte proliferation (*p*<0.05), which tended to decrease at week 12 (*p*<0.10) and linearly decreased at week 24 with increasing vitamin A levels (Table 9) (*p*<0.05).

**Table 9:** Peripheral blood lymphocyte proliferation, liver functions, and mRNA expression of vitamin D receptor (VDR).

Vitamin A Treatment (IU/kg)	Con A		VDR mRNA	AST (U/L)		TBIL (µmol/L)	
	Week 12	Week 24	Week 24	Week 12	Week 24	Week 12	Week 24
A (5,000)	1.14	1.15^ab^	1.46^a^	264.00	261.75	6.14	5.80
B (15,000)	1.19	1.29^b^	1.58^a^	280.17	274.75	5.82	6.55
C (45,000)	1.16	1.16^ab^	2.29^ab^	252.40	267.67	7.40	3.60
D (135,000)	0.82	0.83^a^	2.54^b^	283.00	371.80	4.65	7.03
SEM	0.065	0.063	0.172	7.493	12.082	0.352	0.311
*p*-value							
Combined	0.173	0.075	0.053	0.278	0.105	0.035	0.001
Linear	0.089	0.044	0.704	0.602	0.057	0.246	0.668
Quadratic	0.136	0.052	0.435	0.558	0.206	0.037	0.033

AST =  aspartate amino transferase, TBIL =  total bilirubin.

a–b Within a column, values not sharing a common superscript letter are significantly different (P<0.05).

### Vitamin D receptor mRNA expression

Vitamin D receptor mRNA expression linearly increased with increasing levels of vitamin A in the diet (Table 9) (*p*<0.05).

### Serum biochemistry

In week 12, serum total bilirubin quadratically changed with increasing supplementation of vitamin A, but AST did not change (Table 9). In week 24, AST tended to increase linearly (*p*<0.10) and serum total bilirubin quadratically changed with increasing supplementation of vitamin A (*p*<0.05).

## Discussion

### Laying performance of broiler breeders and liver function

The vitamin A requirement for laying hens recommended by the NRC (1994) was 3,000 IU/kg based on 100 g daily feed intake [18]. However, Coşkun et al. reported that increasing levels of vitamin A (0, 4,000, 12,000, and 24,000 IU/kg of diet) had no significant effect on egg production in laying hens for 72 weeks [5]. Mendonça et al. supplemented with 5,000; 10,000; 15,000; 20,000; and 25,000 IU/kg retinyl acetate in Hy-line white laying hens for 15 weeks, and found no adverse effects on egg weight, egg production, or feed consumption [6]. Their work indicated that supplementing with dietary vitamin A at a level more than eight times that of the NRC (1994) did not affect the laying performance of laying hens. However, March et al. reported that supplementation with higher levels of vitamin A (210,000 and 410,000 IU/kg) significantly decreased egg production and egg weight [10].

In the current study, supplementation of 35,000 IU/kg dietary vitamin A, more than 11 times the level recommended by the NRC (1994) [18], did not affect the laying performance of broiler breeders (data not shown). However, when vitamin A was supplemented at 45,000 IU/kg, 15 times the NRC's recommendation (1994), or at 135,000 IU/kg, 45 times the NRC's recommendation (1994), egg weight was significantly decreased (Table 3), compared with egg weight from birds that received only 5,000 IU/kg of supplemental dietary vitamin A. This result agreed with the findings of the NRC (1987) [4] that vitamin A has certain toxic effects.

Although the NRC (1994) [18] did not recommend a daily requirement of vitamin A for broiler breeders, commercial broiler breeder companies recommend daily supplementation of 10,000 IU/kg [8], and usually a much higher level ofvitamin A is supplemented to broiler or broiler breeder diets. Therefore, we suggest that 35,000 IU/kg is the maximum tolerable dose of vitamin A for broiler breeders. Supplementation with different levels of vitamin A linearly increased vitamin A in the liver and yolk (Tables 6, 7), confirming the results of previous studies that egg retinol increased linearly with increasing dietary vitamin A levels [6], [11], [12], [25]. The liver is the major storage site for vitamin A, containing 80% of total body reserves when vitamin A status is normal [26]. Excessive amounts of vitamin A retained in liver may exert a hepatotoxic effect. McCuaig and Motzok showed that the livers of broilers became light and clay-colored when the birds were fed high doses of 325,0000 IU/kg vitamin A [13].

Serum AST and TBIL are commonly measured clinically during diagnostic liver function tests to determine liver health. The levels of serum AST generally increase with muscle or liver damage [27]. The current study found that excessive dietary vitamin A increased serum AST, and that dietary vitamin A quadratically affected TBIL concentration. The decreased egg weight in birds fed excessive vitamin A might be attributed to the affected liver function.

### Egg quality and hatchability

March et al. found that excessive vitamin A prolonged incubation time, markedly depressed hatchability, and caused developmental abnormalities in embryos, including hemorrhaging that resulted in embryonic death [10]. An excess of vitamin A during embryonic development was also found to result in congenital malformations [1]. In the current study, increasing vitamin levels from 5,000 IU/kg to 35,000 IU/kg did not affect hatchability of eggs. However, supplementing vitamin A at 45,000 IU/kg significantly decreased hatchability from week 24, and supplementing vitamin A at 135,000 IU/kg significantly decreased the hatchability of hatched or fertile eggs from week 12.

The current study found that excessive vitamin A affected concentrations in the yolk and liver of four tocopherols, naturally occurring fat-soluble nutrients. This effect may be related to vitamin absorption in the intestine and metabolism by the liver. Vitamin A decreased the α-tocopherol concentration in yolk and in liver. Previous work found that increased levels of dietary vitamin A had adverse effects on the absorption of vitamin E [11], [12], [28], which includes α-, β-, γ-, δ-tocopherol and four compounds with tocotrienol structure bearing three double bonds in the phytyl side chain (α-,β-,γ-,δ-tocotrienol). Of these, δ-tocopherol has the highest antioxidant effects, and α-tocopherol has the highest biological activity and molar concentration of lipid-soluble antioxidants in animals [29].

It is known that all forms of vitamin E are absorbed in the intestine, but there are several unknowns concerning metabolism. α-tocopherol is absorbed by a passive diffusion process from the small intestine to the enterocyte [30]. Dietary retinoic acid reduced the intestinal absorption of α-tocopherol and promoted its oxidation [31] or increased vitamin E turnover [28]. Research on vitamin E absorption found that excess α-tocopherol did not reduce the absorption of γ-tocopherol [32]. In addition, it is known that the liver preferentially secretes α- but not γ- tocopherol into plasma. γ-tocopherol is metabolized to γ- carboxy ethyl hydroxy chroman [33]. This may be the reason that α-tocopherol and total tocopherol in yolk and liver decreased as vitamin A levels increased; however, δ-tocopherol in yolk and γ- and δ-tocopherol in liver linearly increased with increasing vitamin A levels. These results suggest that we should consider the reduced biological activity and anti-sterility activity caused by α-tocopherol, and improved antioxidant effects caused by δ-tocopherol, when the diet is supplemented with higher level of vitamin A.

The eggshell performs the double function of protecting the embryo from external damage and supplying calcium during embryo development. Decreased eggshell thickness increased early embryonic mortality [34]. Mendonça et al. reported that supplementation of 5,000; 10,000; 15,000; 20,000; or 25,000 IU/kg retinyl acetate had no adverse effects on specific gravity, shell index, or albumen quality of eggs [6]. However, the eggshell thickness of eggs from hens that received 5,000 or 20,000 IU/kg of retinyl acetate was statistically lower than those of a control group.

The current study found that supplementation of 5,000–35,000 IU/kg of vitamin A did not affect eggshell thickness in Experiment 1 (data not shown); however, supplementation of 5,000–135,000 IU/kg of vitamin A in week 24 quadratically affected eggshell thickness (*p*<0.05) and strength in Experiment 2 (*p*<0.10). The eggshell thickness and strength were mainly related to calcification. High levels of vitamin A had a detrimental effect on calcification, and reduced calcium absorption affected eggshell thickness, especially over long-term feeding trials. This effect may be the reason for the difference in egg quality between Experiment1 and Experiment 2.

Veltmann et al. showed that birds tolerated as much as 30 times the recommended level of vitamin A (45,000 IU/kg) without showing compromised performance or skeletal development [16]. However, high levels of dietary vitamin A (45,000 IU/kg) interfered with the utilization of vitamin D_3_, 25-(OH)D_3_ and 1,25-(OH)_2_D_3_, and decreased bone ash (p<0.001) [35]. There is also a correlation between high intake of vitamin A and low bone mineral density [36]. Lind et al. found that excessive vitamin A resulted in a reduction of long bone diameter and increase in spontaneous fractures of rats [37]. High levels of dietary vitamin A also resulted in congenital malformations during embryonic development [1].

Excessive supplementation with vitamin A might increase bone fracture risk by depleting vitamin D [14]. Vitamin D receptor, the transport protein of vitamin D, plays an important role in the absorption of vitamin D. The current study showed that excessive levels of vitamin A increased mRNA expression of vitamin D receptor, which might be a compensatory mechanism for poor vitamin D absorption at the molecular level.

In the current study, yolk color was linearly decreased by increased dietary vitamin A (Table 4). Yolk color is mainly affected by a large component of yellow, fat-soluble pigments, such as carotenes, *β*-carotene, and xanthophylls. The absorption of these fat-soluble pigments, like the absorption of fat-soluble vitamins, might be reduced by a high intake of vitamin A [6], [11], [12].

### Immune function and serum antibody against NDV

Vitamin A also promotes antibody responses to T-cell–dependent antigens [38] and increases protective antitumor immunity through mechanisms such as induction of cell differentiation and enhancement of migration to lymph nodes [39].

Previous work showed that excessive vitamin A intake has a detrimental effect on the immune function of birds [15]. The current study showed that supplementation of vitamin A at 135,000 IU/kg significantly decreased peripheral blood T-cell proliferation activity at week 24. Supplemental levels of vitamin A above the tolerable dose could impair T-cell immunity.

Supplementation of vitamin A from 5,000 IU/kg to 20,000 IU/kg appears to increase the NDV antibody titer, but supplementation from 20,000 to 35,000 IU/kg decreased the NDV antibody titer. Lessard et al. found that NDV antibody titers were significantly increased as laying broiler hens consumed 1,500 IU/kg to 15,000 IU/kg of vitamin A *ad libitum* [40]. Their study indicated that supplementation of excessive vitamin A was detrimental to humoral immunity.

## Conclusions

Excessive supplementation of vitamin A could decrease liver function, reproductive performance, and immune response of broiler breeder hens. The maximum safety tolerable dose of vitamin A for broiler breeders is 35,000 IU/kg.The mechanism of dietary addition of vitamin A affected the α-, γ-, and total tocopherol concentrations in yolk or in liver requires further study.

## References

[1] Clagett-Dame M, DeLuca HF (2002) The role of vitamin A in mammalian reproduction and embryonic development. Annu Rev Nutr 22: 347–381. 12055350 10.1146/annurev.nutr.22.010402.102745E

[2] Davis CY, Sell JL (1983) Effect of all-transretinol and retinoic acid nutriture on the immune system of chicks. J Nutr 113: 1914–1919. 6619972 10.1093/jn/113.10.1914

[3] Sklan D, Melamed D, Friedman A (1994) The effect of varying levels of dietary vitamin A on Immune response in the Chick. Poult Sci 73: 843–847. 8072927 10.3382/ps.0730843

[4] NRC (1987) Vitamin Tolerance of Animals. National Academy Press, Washington, DC.

[5] Coşkun B, Inal F, Celik I, Erganis O, Tiftik AM, et al (1998) Effects of dietary levels of vitamin A on the egg yield and immune responses of laying hens. Poult Sci 77: 542–546. 9565236 10.1093/ps/77.4.542

[6] MendonçaJr CX, Almeida CRM, Mori AV, Watanabe C (2002) Effect of dietary vitamin A on egg yolk retinol and tocopherol levels. J Appl Poult Res 11: 373–378.

[7] Lin H, Wang LF, Song JL, Xie YM, Yang QM (2002) Effect of dietary supplemental levels of vitamin A on the egg production and immune responses of heat-stressed laying hens. Poult Sci 81: 458–465. 11989744 10.1093/ps/81.4.458

[8] ROSS 308 Broiler Nutrition Specification June 2007. Aviagen Incorporated. Cummings Reaesrch Park, 5015, Bradford Drive Huntsville, Alabama 35805, USA

[9] O'Byrne SM, Blaner WS (2013) Retinol and retinyl esters: biochemistry and physiology. J Lipid Res 54: 1731–1743. 23625372 10.1194/jlr.R037648 PMC3679378

[10] March BE, Coates V, Goudie C (1972) Delayed hatching time of chicks from dams fed excess vitamin A and from eggs injected with vitamin A. Poult Sci 51: 891–896. 4646669 10.3382/ps.0510891

[11] Jiang YH, McGeachin RB, Bailey CA (1994) α-Tocopherol, β-carotene, and retinol enrichment of chicken eggs. Poult Sci 73: 1137–1143. 7937475 10.3382/ps.0731137

[12] Surai PF, Ionov IA, Kuklenko TV, Kostjuk IA, Macpherson A, et al (1998) Effect of supplementing the hen's diet with vitamin A on the accumulation of vitamins A and E, ascorbic acid and carotenoids in the egg yolk and in the embryonic liver. Br Poult Sci 39: 257–263. 9649881 10.1080/00071669889222

[13] McCuaig LW, Motzok I (1970) Excessive dietary vitamin E: its alleviation of hypervitaminosis A and lack of toxicity. Poult Sci 49: 1050–1052. 5485475 10.3382/ps.0491050

[14] Caire-Juvera G, Ritenbaugh C, Wactawski-Wende J, Snetselaar LG, Chen Z (2009) Vitamin A and retinol intakes and the risk of fractures among participants of the Women's Health Initiative Observational Study. Am J Clin Nutr 89: 323–330. 19056568 10.3945/ajcn.2008.26451 PMC2715292

[15] Freidman A, Meidovaky A, Leitner G, Sklan D (1991) Decreased resistance and immune response to Escherichia coli infection in chicks with low or high intakes of vitamin A. J Nutr 121: 395–400. 2002410 10.1093/jn/121.3.395

[16] Veltmann JR, Jensen LS, Rowland GN (1986) Excess dietary vitamin A in the growing chick: effect of fat source and vitamin D. Poult Sci 65: 153–163. 3960810 10.3382/ps.0650153

[17] Veltmann JR, Jensen LS (1986) Vitamin A Toxicosis in the Chick and Turkey Poults. Poult Sci 65: 538–545. 3703795 10.3382/ps.0650538

[18] National Research Council (1994) Nutrient Requirement of Poultry. 9th ed. National Academy Press, Washington, DC.

[19] Allan WH, Gough RE (1974) A standard haemagglutination inhibition test for Newcastle disease. (1). A comparison of macro and micro methods. Vet Rec 95: 120–123. 4446306 10.1136/vr.95.6.120

[20] Boily MH, Champoux L, Bourbonnais DH, Desgranges JL, Rodreigue J (1994) Beta-carotene and retinoids in eggs of great Blue Herons (Ardeaherodias) in relation to St. Lawrence River contamination. Ecotox 3: 271–286. 10.1007/BF00117992 24202125

[21] Catignani GL, Driskell WJ, Bashor MM, Turley CP, Brewster MA (1983) Simultaneous determination of retinol and a-tocopherol in serum or plasma by liquid chromatography. Clin Chem 29: 708–712. 6831704

[22] Zhang LB, Guo YM (2008) Effects of liquid dl-2-Hydroxy-4-Methylthio butanoic acid on growth performance and immune responses in broiler chickens. Poult Sci 87: 1370–1376. 18577618 10.3382/ps.2007-00366

[23] Pfaffl MW (2001) A new mathematical model for relative quantificationin real-time RT-PCR. Nucleic Acids Res 29: e45. 11328886 10.1093/nar/29.9.e45 PMC55695

[24] Spss 15.0 Command Syntax Reference 2006, SPSS Inc., Chicago Ill

[25] Livingston KA, Klasing KC (2011) Retinylpalmitate does not have an adjuvant effect on the antibody response of chicks to keyhole limpet hemocyanin regardless of vitamin A status. Poult Sci 90: 965–970. 21489940 10.3382/ps.2010-01085

[26] Blomhoff R, Wake K (1991) Perisinusoidal stellate cells of the liver: important roles in retinol metabolism and fibrosis. FASEB J 5: 271–277. 2001786 10.1096/fasebj.5.3.2001786

[27] Sohail MU, Rahman ZU, Ijaz A, Yousaf MS, Ashraf K, et al (2011) Single or combined effects of mannan-oligosaccharides and probiotic supplements on the total oxidants, total antioxidants, enzymatic antioxidants, liver enzymes, and serum trace minerals in cyclic heat-stressed broilers. Poult Sci 90: 2573–2577. 22010243 10.3382/ps.2011-01502

[28] Sklan D, Donoghue S (1972) Vitamin E response to high dietary vitamin A in the chick. J Nutr 112: 759–765. 10.1093/jn/112.4.759 7069511

[29] Drevon CA (1991) Absorption, transport and metabolism of vitamin E. Free Radic Res Commun 14: 229–246. 1874454 10.3109/10715769109088952

[30] Krishnan K, Campbell S, Stone WL (2005) High-dosage vitamin E supplementation and all-cause mortality. Ann Intern Med 143 151; author reply 156–158. 10.7326/0003-4819-143-2-200507190-00019 16027462

[31] Bieri JG, Wu AL, Tolliver TJ (1981) Reduced intestinal absorption of vitamin E by low dietary levels of retinoic acid in rats. J Nutr 111: 458–467. 7205402 10.1093/jn/111.3.458

[32] Leonard SW, Paterson E, Atkinson JK, Ramakrishnan R, Cross CE, et al (2005) Studies in humans using deuterium-labeled α- and γ-tocopherol demonstrate faster plasma γ-tocopherol disappearance and greater γ-metabolite production. Free Radic Biol Med 38: 857–866. 15749381 10.1016/j.freeradbiomed.2004.12.001

[33] MacMahon MT, Neale G (1970) The absorption of alpha-tocopherol in control subjects and in patients with intestinal malabsorption. Clin Sci 38: 197–210. 5416150 10.1042/cs0380197

[34] Mroz E, Michalak K, Orlowska A (2007) Embryo mortality and poult quality depend on the shell structur of turkey hatching eggs. Anim Sci and Reports 25: 161–172.

[35] Aburto A, Edwards JR HM, Britton WM (1998) The influence of vitamin A on the utilization and amelioration of toxicity of cholecalciferol, 25-Hydroxycholecalciferol, and 1,25 Dihydroxycholecalciferol in young broiler chickens. Poult Sci 77: 585–593. 9565243 10.1093/ps/77.4.585

[36] Forsmo S, Fjeldbo SK, Langhammer A (2008) Childhood cod liver oil consumption and bone mineral density in a population-based cohort of peri- and postmenopausal women: The nord-trøndelag health study. Am J Epidemiol 167: 406–411. 18033763 10.1093/aje/kwm320

[37] Lind T, Sundqvist A, Hu L, Pejler G, Andersson G, et al (2013) Vitamin a is a negative regulator of osteoblast mineralization. PLoS One(12): e82388 doi:10.1371/journal.pone.0082388 10.1371/journal.pone.0082388 PMC3858291 24340023

[38] Catharine RA (2012) Vitamin A and retinoic acid in T cell–related immunity. Am J Clin Nutr 96 suppl: 1166S–1172S. 23053562 10.3945/ajcn.112.034637 PMC3471201

[39] Mullin GE (2011) Vitamin A and Immunity. Nutr Clin Pract 26: 495–496. 21775644 10.1177/0884533611411583

[40] Lessard M, Hutchings D, Cave NA (1997) Cell-mediated and humoral immune responses in broiler chickens maintained on diets containing different levels of vitamin A. Poult Sci 76: 1368–137. 9316112 10.1093/ps/76.10.1368

